# Involvement of Endothelin 1 in Remote Preconditioning-Induced Cardioprotection through connexin 43 and Akt/GSK-3β Signaling Pathway

**DOI:** 10.1038/s41598-018-29196-x

**Published:** 2018-07-19

**Authors:** Min Zhang, Wei Wei Gu, Xing yu Hong

**Affiliations:** 1 Hepatobiliary pancreatic surgery, China-Japan Union Hospital of Jilin University, 126 XianTaiStreet, Changchun, 130033 China; 2 Vascular surgery, China-Japan Union Hospital of Jilin University, 126 XianTai Street, Changchun, 130033 China

## Abstract

The present study was aimed to explore the role of endothelins in remote preconditioning (RP)-induced myocardial protection in ischemia-reperfusion (IR) injury. RP stimulus was given by subjecting hind limb to four cycles of ischemia and reperfuion (5 minutes each) using blood pressure cuff in male rats. Following RP, hearts were isolated and subjected to 30 minutes of ischemia and 120 minutes of reperfusion on Langendorff apparatus. The extent of myocardial injury was determined by measuring the levels of LDH-1, CK-MB and cardiac troponin T (cTnT) in coronary effluent; caspase-3 activity and Bcl 2 expression in heart (apoptosis); infarct size by triphenyl tetrazolium chloride and contractility parameters including left ventricular developed pressure, dp/dt_max_ dp/dt_min_ and heart rate. RP reduced ischemia reperfusion-induced myocardial injury, increased the levels of endothelin 1 (in blood), Akt-P, GSK-3β-P and P-connexin 43 (in hearts). Pretreatment with ET_A_ receptor antagonist, BQ 123 (1 and 2 mg/kg), ET_B_ receptor antagonist, BQ 788 (1 and 3 mg/kg) and dual inhibitor of ET_A_ and ET_B_ receptor, bonsentan (25 and 50 mg/kg) abolished these effects of RP. However, the effects of bonsentan were more pronounced in comparison to BQ 123 and BQ 788. It is concluded that RP stimulus may release endothelin 1 in the blood, which may activate myocardial ET_A_ and ET_B_ receptors to trigger cardioprotection through connexin 43 and Akt/GSK-3β pathway.

## Introduction

Remote preconditioning is a novel strategy to protect the heart from the harmful effects of prolonged ischemia and reperfusion (I/R) by subjecting a remote organ (other than heart) to short cycles of ischemia and reperfusion^1,2^. In ischemic preconditioning the preconditioning stimulus (short episodes of ischemia and reperfusion) is delivered to heart itself before prolonged ischemia and reperfusion^3,4^. There have been a number of studies showing the utility of remote preconditioning in reducing ischemic injury to heart in animals^5,6^ as well as in humans^2,7^. Nevertheless, the mechanisms involved in remote preconditioning-induced myocardial protection are still not fully elucidated. Therefore, the present study has been designed to reveal the mechanisms involved in inducing cardioprotection during remote preconditioning.

According to the humoral theory of remote preconditioning, short episodes of ischemia and reperfusion may induce the release of some factors from the remote organ, which are conveyed by the blood to the heart to trigger cardioprotective signaling^8^. It may also be possible that the blood vessels in the remote organ region respond to short episodes of ischemia and reperfusion by releasing cardioprotective humoral factors. The role of blood vessels as the potential source of cardioprotective factors is supported by the studies showing comparable degree of cardioprotection in remote renal preconditioning, remote hind limb preconditioning, remote aortic preconditioning and remote mesenetric preconditioning^9–12^. Indeed, short episodes of ischemia to kidney, hind limb, intestine or liver produce cardioprotection to a comparable extent suggesting that the blood vessels may be common tissue in these organs to release cardioprotective factors.

Endothelium is the innermost lining of the blood vessels and it is very sensitive to local changes in blood flow. It responds very quickly by releasing endothelial-derived factors in response to a number of stimuli such as ischemia^13–15^. Endothelin is one of the important endothelial derived factors and it exerts a large number of biological responses. The endothelin family comprises of 21-amino-acid long isopeptides i.e., endothelin-1, endothelin-2 and endothelin-3. Amongst these peptides, endothelin 1 is widely distributed in the cardiovascular system and it is primarily referred as a vasoconstrictive peptide^16,17^. In mammals, these endothelins act on the endothelin receptors, ET_A_ and ET_B_^18^, which are present on the different body parts including heart^19^. Endothelin 1 and endothelin 2 activate ET_A_ receptors to a comparable degree; however, endothelin 3 does not activate ET_A_ receptors. On the other hand, all endothelins have same affinity for ET_B_ receptors^20^.

It is interesting to note that the stimuli or agents that produce myocardial injury are reported to precondition the heart. Ischemia^3^, free radicals^21^ and calcium^22^ are well documented to produce myocardial injury. However, there has been a large of studies showing that these agents also precondition the heart and protect it from subsequent sustained ischemic injury^3,21,22^. Similarly, there have been studies suggesting that endothelin may contribute in inducing myocardial injury during ischemia and reperfusion^23,24^. On the other hand, exogenous administration of endothelin has also been shown to produce preconditioning like effects in heart^25,26^. Accordingly, it is hypothesized that the endothelium of the blood vessels of the remote organ may respond to short episodes to ischemia by releasing endothelins, which may act as cardioprotective humoral factors. However, the role of endothelins in remote ischemic preconditioning-induced cardioprotection is not explored yet. Therefore, the present was performed to explore the role of endothelins in remote preconditioning-induced myocardial protection in ischemia reperfusion injury in rats.

## Material and Methods

### Animals, Chemicals and Drugs

Wistar albino male rats (250–300 g) were employed in the study and rats were purchased from Jilin University. The animal studies were approved by the Institutional Review Board and Institutional Animal Care and Use Committee of the Jilin University, and all of the experiments were performed in accordance with the approved protocols. BQ-123, a selective ET_A_ receptor antagonist, and BQ788, a selective ET_B_ receptor antagonist, were procured from EMD Bioscience, USA. Bonsentan, a dual blocker of ET_A_ and ET_B_ receptors, was procured from Sigma-Aldrich, USA. The diagnostic kits for quantitative estimation of LDH-1, CK-MB and cardiac troponin T were procured from Jiancheng Reagent Co, Nanjing, China. ELISA kits for quantitative estimation of endothelins, caspase 3, bcl-2, phosphorylated GSK-3β, Akt and connexin 43 were procured from Life BioSpan Biosciences, USA. BQ-123 was solubilized in water, while BQ788 and bonsentan were dissolved in DMSO. The doses of BQ 123^27^, BQ 788^28^ and bonsentan^29^ were selected the basis of published literature.

### Remote Preconditioning and Isolated Heart preparation of Langendorff System

Thiopentone (35 mg kg^−1^, *i*.*p*.) was used to anesthetize the animals and left hind limb was tied with a blood pressure cuff at the inguinal level. The cuff was alternatively inflated (up to 150 mm of Hg) and deflated for 5 minutes to induce ischemia and reperfusion, respectively. Four such episodes (each episode consisting of 5 minutes ischemia and 5 minutes reperfusion) constituted remote preconditioning stimuli^30^. After last episode of remote preconditioning, rat was sacrificed and heart was isolated to perfuse with physiological solution (Kreb’s Henseleit solution) on the Langendorff system^31^. Global ischemia was produced for 30 minutes by blocking perfusion to the heart and reperfusion was given for 120 minutes by restoring perfusion to the heart. A balloon, connected to catheter and pressure transducer, was inserted in the left ventricle to record contractility parameters (mentioned below). The coronary perfusate was collected at different time intervals to measure the biochemical parameters and assess the extent of myocardial injury.

### Determination of Biochemical Parameters of Heart Injury

The injury to heart was assessed by measuring the release of specific biomarkers from the heart into the coronary perfusate. The measurement of LDH-1, CK-MB and cardiac troponins (cTnT) were done using commercial kits. The release of LDH-1 from heart was determined by measuring its levels in the coronary perfusate immediately before global ischemia and 30 minutes after reperfusion. The levels of CK-MB and cTnT were measured in coronary effluent before global ischemia and immediately after reperfusion.

### Determination of Myocardial Infarct Size

The extent of heart injury and its modulation with remote preconditioning was determined macroscopically by determining myocardial infarction with triphenyltetrazolium chloride (TTC) staining. TTC staining is one of the standard methods of assessing cell viability. TTC stains viable cells red, while dead cells remain unstained. In this method, heart was cut in thin slices and stained with TTC. Thereafter, area of infarcted (unstained) and viable portions (stained) were assessed using planimetry^32^.

### Measurement of Functional Parameters of Heart

A balloon, connected to catheter and pressure transducer, was inserted in the left ventricles of rat hearts to determine heart contractility including left ventricular developed pressure (LVDP), dp/dt_max_ (rate of contraction), dp/dt_min_ (rate of relaxation) and heart rate. These contractility parameters were measured throughout the experiment and comparison was made between pre-ischemic and post-ischemic contractility parameters.

### Determination of Endothelin levels in the Plasma

After last episode of remote ischemic stimulus, the blood was removed from rats and the plasma was separated by centrifugation. In ischemia reperfusion group, blood was withdrawn immediately before heart isolation. The levels of endothelin 1, endothelin 2 and endothelin 3 in the plasma were measured using commercially available ELISA kits.

### Determination of Apoptosis Markers, GSK-3β-P, Akt-P and P-connexin 43 in the Heart

After global ischemia-reperfusion on the Langendorff apparatus, the hearts were isolated. The half portion of the heart was homogenized in the lysis solution containing NP-4 and protease inhibitor. The other half portion of the heart was used to measure myocardial infarction using TTC staining. The homogenized mixture was centrifuged and the supernatant was used to measure the levels of p-GSK-3β-S9, Akt-P, P-connexin 43, bcl-2 along with caspase 3 activity using commercially available ELISA kits. Caspase 3 activity was standardized with respect to total protein content^33^.

### Experimental Groups

Animals were distributed in various groups (n = 6) to meet the objectives of study and these included: ***i***. ***Ischemia reperfusion injury***: Isolated rat hearts were subjected to thirty minutes of ischemia and one twenty minutes of reperfusion on the Langendorff apparatus; ***ii***. ***Remote preconditioning***: Four episodes of ischemia and reperfusion (each of five minutes) were given in rats before prolonged ischemia and reperfusion on the Langendorff apparatus; ***iii***. ***and iv***. ***BQ-123***(***1 and 2 mg/kg i***.***p***.) ***in remote preconditioning***: BQ-123 was administered in rats thirty minutes before remote preconditioning; ***v***. ***and vi BQ788*** (***1 and 3 mg/kg i***.***p***.) ***in remote preconditioning***: BQ-788 was administered in rats thirty minutes before remote preconditioning; ***vii***. ***and viii***. ***Bonsentan*** (***25 and 50 mg/kg i***.***p***.) ***in remote preconditioning***: Bonsentan was administered in rats thirty minutes before remote preconditioning; ***ix***. ***DMSO*** (***0***.***5 ml/kg***) ***in remote preconditioning***: 10% DMSO (solvent of BQ 788 and bonsentan) was administered in rats thirty minutes before remote preconditioning; ***x***. ***Normal Rats***: Neither treatment nor ischemia reperfusion was given to animals. The hearts were isolated to measure the basal levels of GSK3β-P, Akt-P, P-connexin 43, bcl-2 and caspase 3 activity.

### Statistical Analysis

The data were presented in the form of mean ± standard deviation (S.D.). Two-way ANOVA was employed to compare the data of LDH-1, CK-MB, cTnT, LVDP, +dp/dt_max_, −dp/dt_min,_ and heart rate. One-way ANOVA was used to compare the data of infarct size, P-Akt, P-GSK-3β, P-connexin 43, bcl-2 and caspase 3. Unpaired t test was used to analyse the data of endothelin levels. Tukey’s test was employed for *post hoc* analysis. Statistical significance was calculated by fixing p < 0.05.

## Results

### Ischemia reperfusion produced injury to isolated rat hearts

**Figure 1:**
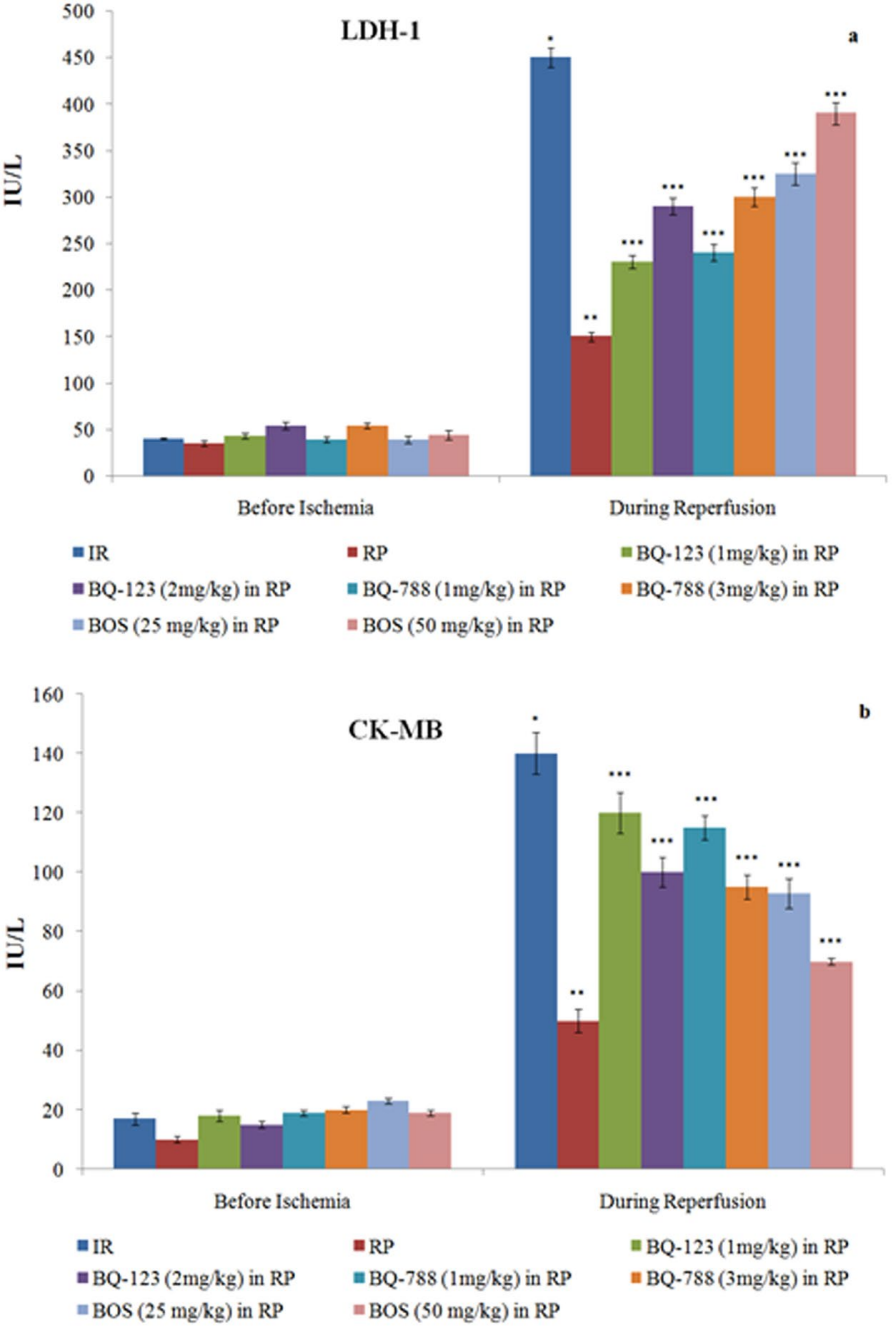
Consequences of I/R, RP and endothelin receptor antagonists on (**a**) LDH-1 release, measured before ischemia and 30 minutes after reperfusion; (**b**) CK-MB release, measured before ischemia and immediately after reperfusion. *p < 0.05 *vs*. before ischemia; **p < 0.05 *vs*. I/R; ***p < 0.05 *vs*. RP. Two way ANOVA was followed by Tukey’s test and statistic parameters for LDH-1 were n = 6, F(1, 80) = 1004.1, p < 0.0001 for time; F(7, 80) = 450.5, p < 0.0001 for treatment and F(7, 80) = 340.5, p < 0.0001 for interaction. Statistic parameters for CK-MB were n = 6, F(1, 80) = 799.1, p < 0.0001 for time; F(7, 80) = 359.5, p < 0.0001 for treatment and F(7, 80) = 270.1, p < 0.0001 for interaction.

**Figure 2:**
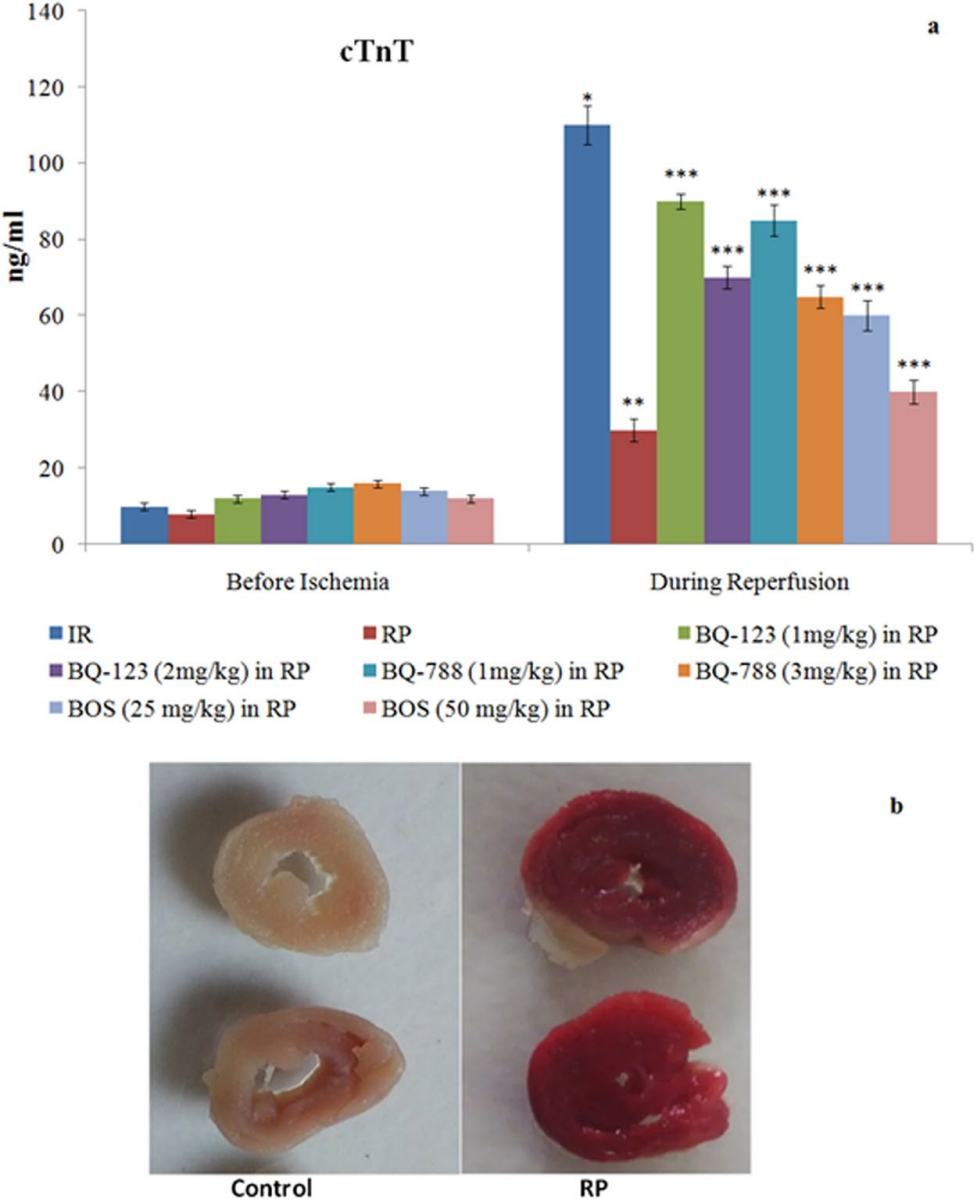
(**a**) Consequences of I/R, RP and endothelin receptor antagonists on cTnT release in coronary effluent, measured before ischemia and immediately after reperfusion. *p < 0.05 *vs*. before ischemia; **p < 0.05 *vs*. I/R; ***p < 0.05 *vs*. RP. Two way ANOVA was followed by Tukey’s test and statistic parameters for cTnT were n = 6, F(1, 80) = 679.1, p < 0.0001 for time; F(7, 80) = 344.5, p < 0.0001 for treatment and F(7, 80) = 220.5, p < 0.0001 for interaction. (**b**) depicts the representative pictures of hearts following TTC staining. Non-stained heart slices represent infarcted portions, while stained portions represent viable portions of heart.

**Figure 3:**
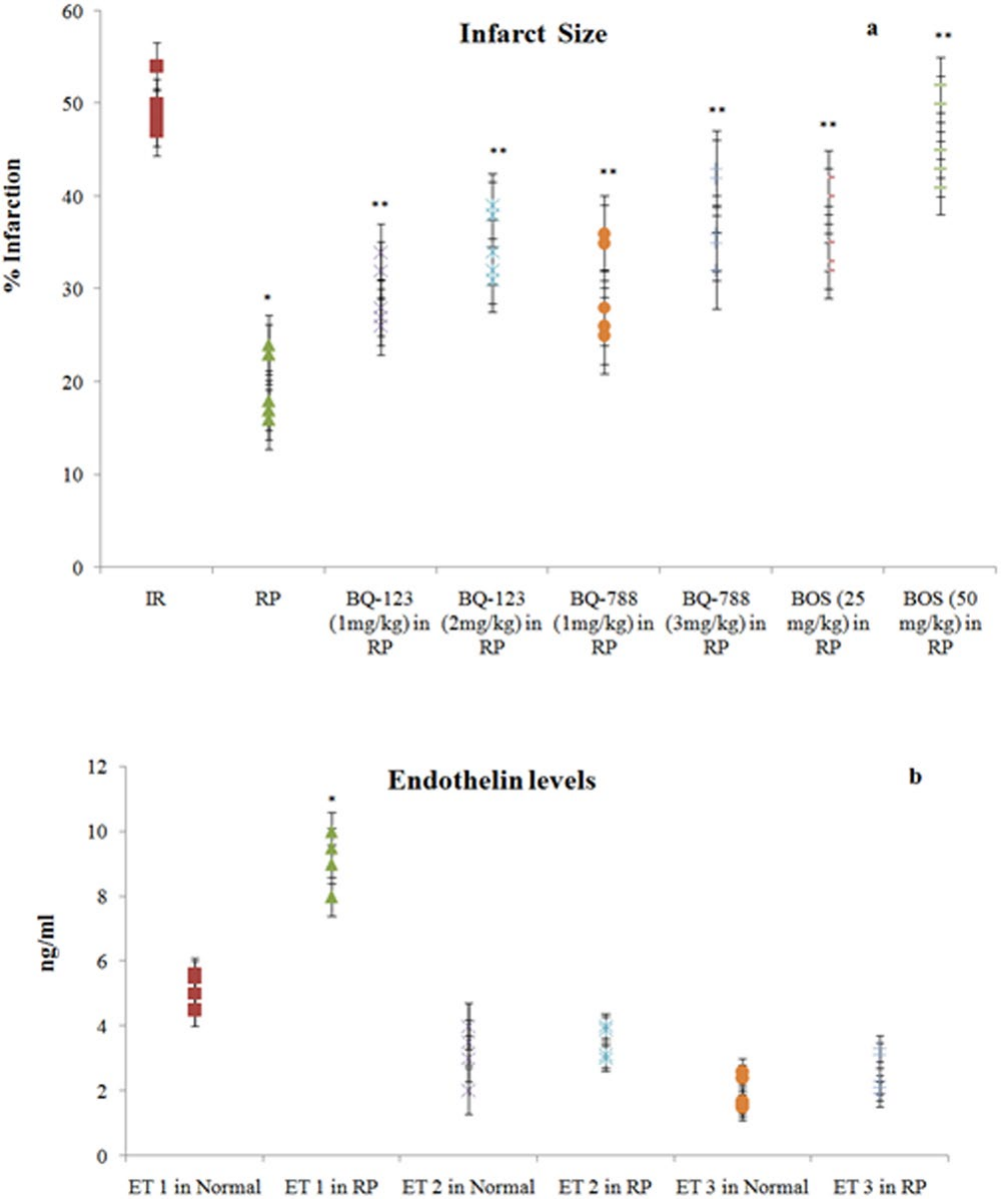
Consequences of I/R, RP and endothelin receptor antagonists on (**a**) infarct size measured by TTC staining; 3(**b**) endothelin levels in coronary effluent, measured immediately before sacrificing rats. *p < 0.05 *vs*. I/R; **p < 0.05 *vs*. RP. For infarction, one way ANOVA was followed by Tukey’s test and statistic parameters were n = 6, F(7, 40) = 304.7, p < 0.0001. For endothelin levels, unpaired two tail t-test was applied to compare ET levels in normal and RP: For ET1, n = 6, t = 9.204, df = 10, p < 0.0001; For ET2, n = 6, t = 1.746, df = 10, p = 0.1114; For ET3, n = 6, t = 1.464, df = 10, p = 0.1739.

**Figure 4:**
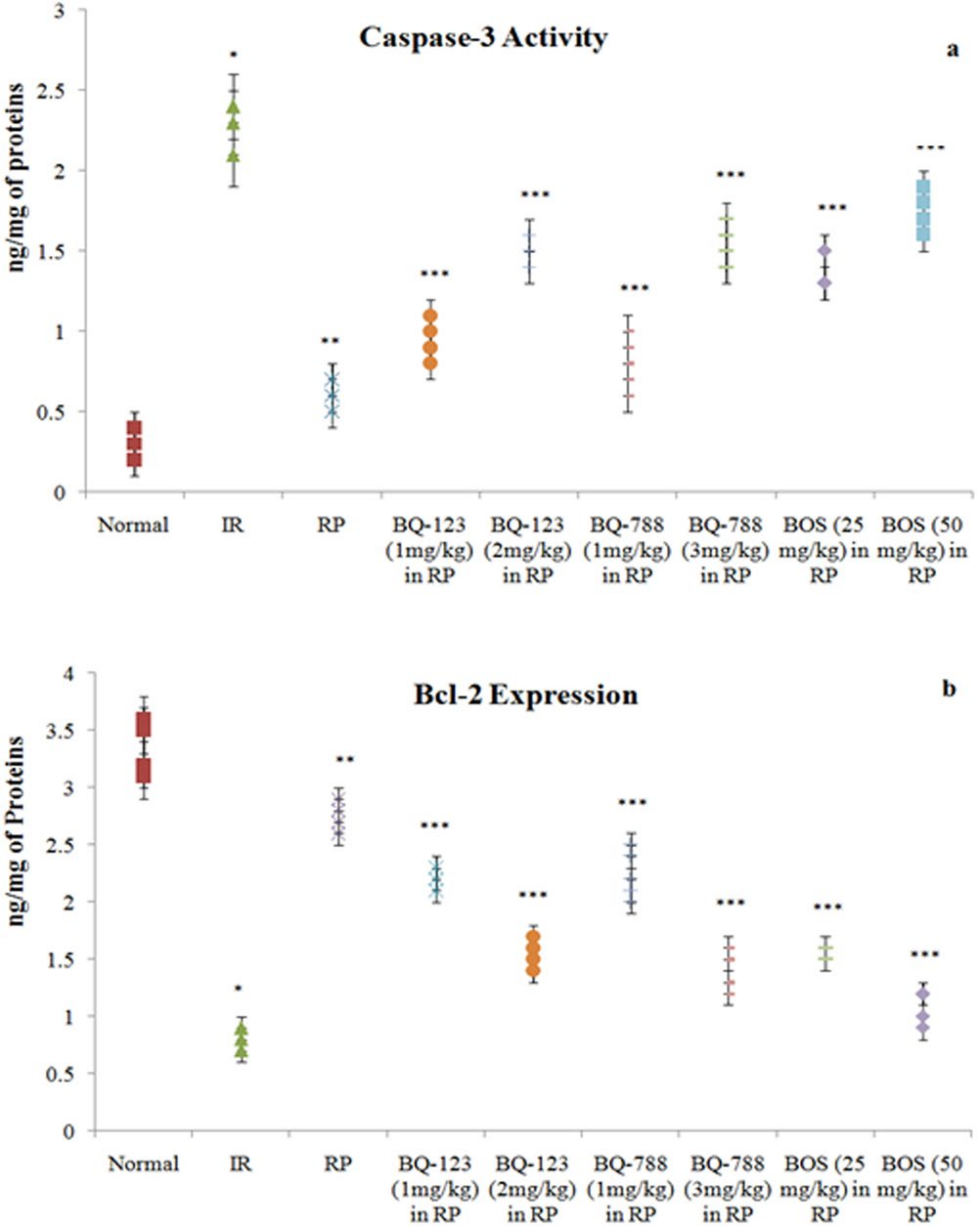
Consequences of I/R, RP and endothelin receptor antagonists on 4(**a**) caspase-3 activity and Bcl-2 expression in heart. *p < 0.05 *vs*. normal; **p < 0.05 *vs*. I/R; ***p < 0.05 *vs*. RP. One way ANOVA was followed by Tukey’s test and statistic parameters for caspase-3 activity were n = 6, F(8, 45) = 232.1, p < 0.0001 and for Bcl-2 expression were n = 6, F(8, 45) = 319.9, p < 0.0001.

In isolated rat hearts, prolonged ischemia followed by reperfusion produced marked injury to heart as evident by an increase in LDH-1 levels in the coronary perfusate at 30 minutes of reperfusion in comparison to basal levels (before ischemia) **(**Fig. 1a**)**. Similarly, there was an increase in the levels of other biochemical markers including CK-MB and cTnT in the coronary perfusate during reperfusion period in comparison to basal levels (before ischemia) **(**Figs 1b and 2a**)**. Moreover, in ischemia-reperfusion subjected hearts, there was a marked decrease in TTC staining area suggesting the significant rise in dead tissue area or myocardial infarction **(**Figs 2b and 3a**)**. Ischemia-reperfusion injury triggered apoptosis as depicted by increase in caspase-3 activity and decrease in bcl-2 expression in hearts **(**Fig. 4a,b**)**. The functional studies also depicted a marked increase in myocardial injury due to prolonged episodes of ischemia and reperfusion. In the present study, there was a marked reduction in heart contractility parameters including LVDP, dp/dt_max_ and dp/dt_min_ during reperfusion phase in comparison to pre-ischemic phase **(**Tables 1, 2 and 3**)**. Moreover, prolonged ischemia and reperfusion also reduced heart rate in a significant manner during reperfusion period **(**Table 4**)**.

**Table 1:** Consequences of I/R, RP and endothelin receptor antagonists on LVDP (mm of Hg).

Experimental Groups	Before Ischemia	During Reperfusion		
		5 minutes	60 minutes	120 minutes
Ischemia reperfusion injury	85 ± 10	30 ± 6*	28 ± 5*	29 ± 5*
Remote Preconditioning	88 ± 11	66 ± 6*^,#^	63 ± 5*^,#^	61 ± 6*^,#^
BQ-123 (1 mg/kg) in remote preconditioning	82 ± 8	55 ± 5*^,≠^	51 ± 6*^,≠^	50 ± 5*^,≠^
BQ-123 (2 mg/kg) in remote preconditioning	89 ± 12	46 ± 7*^,≠^	43 ± 3*^,≠^	41 ± 6*^,≠^
BQ788 (1 mg/kg) in remote preconditioning	80 ± 7	52 ± 3*^,≠^	49 ± 5*^,≠^	47 ± 7*^,≠^
BQ788 (3 mg/kg) in remote preconditioning	84 ± 9	40 ± 5*^,≠^	42 ± 6*^,≠^	39 ± 8*^,≠^
Bonsentan (25 mg/kg) in remote preconditioning	82 ± 9	41 ± 4*^,≠^	42 ± 9*^,≠^	38 ± 5*^,≠^
Bonsentan (50 mg/kg) in remote preconditioning	86 ± 8	32 ± 5*^,≠^	30 ± 6*^,≠^	31 ± 3*^,≠^

*p < 0.05 *vs*. before ischemia.

^#^*p* < 0.05 *vs*. I/R; ^≠^*p* < 0.05 *vs*. RP. I^/^R^=^Ischemia-reperfusion; RP: Remote Preconditioning. Two way ANOVA was followed by Tukey’s test and statistic parameters were n = 6, F(3, 160) = 1509.1, p < 0.0001 for time; F(7, 160) = 650.5, p < 0.0001 for treatment and F(21, 160) = 211.2, p < 0.0001 for interaction.

**Table 2:** Consequences of I/R, RP and endothelin receptor antagonists on dp/dt_max_ (mm of Hg/s).

Experimental Groups	Before Ischemia	During Reperfusion			
		5 minutes	60 minutes		120 minutes
Ischemia reperfusion injury	3521 ± 139	1416 ± 106*		1324 ± 116*	1307 ± 101*
Remote Preconditioning	3622 ± 101	3009 ± 98*^,#^		2946 ± 80*^,#^	2914 ± 99*^,#^
BQ-123 (1 mg/kg) in remote preconditioning	3459 ± 97	2730 ± 75*^,≠^		2666 ± 51*^,≠^	2684 ± 79*^,≠^
BQ-123 (2 mg/kg) in remote preconditioning	3587 ± 105	2243 ± 87*^,≠^		2301 ± 80*^,≠^	2204 ± 59*^,≠^
BQ788 (1 mg/kg) in remote preconditioning	3651 ± 69	2612 ± 61*^,≠^		2505 ± 49*^,≠^	2516 ± 76*^,≠^
BQ788 (3 mg/kg) in remote preconditioning	3437 ± 89	2169 ± 56*^,≠^		2203 ± 69*^,≠^	2196 ± 88*^,≠^
Bonsentan (25 mg/kg) in remote preconditioning	3386 ± 107	2112 ± 56*^,≠^		2038 ± 70*^,≠^	2132 ± 77*^,≠^
Bonsentan (50 mg/kg) in remote preconditioning	3690 ± 98	1781 ± 25*^,≠^		1647 ± 61*^,≠^	1665 ± 86*^,≠^

*p < 0.05 *vs*. before ischemia.

^#^*p* < 0.05 *vs*. I/R; ^≠^*p* < 0.05 *vs*. RP. Two way ANOVA was followed by Tukey’s test and statistic parameters were n = 6, F(3, 160) = 1438.5, p < 0.0001 for time; F(7, 160) = 540.5, p < 0.0001 for treatment and F(21, 160) = 198.2, p < 0.0001 for interaction.

**Table 3:** Consequences of I/R, RP endothelin receptor antagonists on dp/dt_min_ (mm of Hg/s).

Experimental Groups	Before Ischemia	During Reperfusion		
		5 minutes	60 minutes	120 minutes
Ischemia reperfusion injury	3224 ± 119	1219 ± 96*	1123 ± 116*	1104 ± 91*
Remote Preconditioning	3427 ± 81	2809 ± 98*^,#^	2740 ± 70*^,#^	2710 ± 90*^,#^
BQ-123 (1 mg/kg) in remote preconditioning	3357 ± 92	2531 ± 70*^,≠^	2465 ± 53*^,≠^	2488 ± 76*^,≠^
BQ-123 (2 mg/kg) in remote preconditioning	3386 ± 95	2046 ± 81*^,≠^	2105 ± 83*^,≠^	2014 ± 50*^,≠^
BQ788 (1 mg/kg) in remote preconditioning	3454 ± 59	2416 ± 41*^,≠^	2316 ± 53*^,≠^	2317 ± 66*^,≠^
BQ788 (3 mg/kg) in remote preconditioning	3235 ± 80	1960 ± 56*^,≠^	2045 ± 43*^,≠^	1996 ± 80*^,≠^
Bonsentan (25 mg/kg) in remote preconditioning	3187 ± 97	1912 ± 46*^,≠^	1838 ± 56*^,≠^	1939 ± 65*^,≠^
Bonsentan (50 mg/kg) in remote preconditioning	3460 ± 108	1589 ± 56*^,≠^	1440 ± 69*^,≠^	1465 ± 80*^,≠^

*p < 0.05 *vs*. before ischemia.

^#^*p* < 0.05 *vs*. I/R; ^≠^*p* < 0.05 *vs*. RP. Two way ANOVA was followed by Tukey’s test and statistic parameters were n = 6, F(3, 160) = 1201.1, p < 0.0001 for time; F(7, 160) = 451.5, p < 0.0001 for treatment and F(21, 160) = 178.2, p < 0.0001 for interaction.

**Table 4:** Consequences of I/R, RP and endothelin receptor antagonists on heart rate (beats/min).

Experimental Groups	Before Ischemia	During Reperfusion		
		5 minutes	60 minutes	120 minutes
Ischemia reperfusion injury	280 ± 29	120 ± 16*	129 ± 21*	125 ± 18*
Remote Preconditioning	291 ± 31	203 ± 22*^,#^	217 ± 25*^,#^	211 ± 20*^,#^
BQ-123 (1 mg/kg) in remote preconditioning	288 ± 24	190 ± 17*^,≠^	195 ± 14*^,≠^	192 ± 15*^,≠^
BQ-123 (2 mg/kg) in remote preconditioning	278 ± 35	171 ± 29*^,≠^	179 ± 20*^,≠^	175 ± 22*^,≠^
BQ788 (1 mg/kg) in remote preconditioning	284 ± 30	187 ± 26*^,≠^	197 ± 23*^,≠^	190 ± 29*^,≠^
BQ788 (3 mg/kg) in remote preconditioning	277 ± 29	166 ± 26*^,≠^	172 ± 22*^,≠^	170 ± 18*^,≠^
Bonsentan (25 mg/kg) in remote preconditioning	281 ± 27	169 ± 26*^,≠^	172 ± 28*^,≠^	171 ± 27*^,≠^
Bonsentan (50 mg/kg) in remote preconditioning	287 ± 19	146 ± 15*^,≠^	151 ± 18*^,≠^	149 ± 20*^,≠^

*p < 0.05 *vs*. before ischemia; ^#^*p* < 0.05 *vs*. I/R; ^≠^*p* < 0.05 *vs*^.^ RP. Two way ANOVA was followed by Tukey’s test and statistic parameters were n = 6, F(3, 160) = 1101.1, p < 0.0001 for time; F(7, 160) = 310.5, p < 0.0001 for treatment and F(21, 160) = 166.2, p < 0.0001 for interaction.

### Ischemia reperfusion altered the levels of GSK-3β-P, Akt-P and P-connexin 43 in hearts

**Figure 5:**
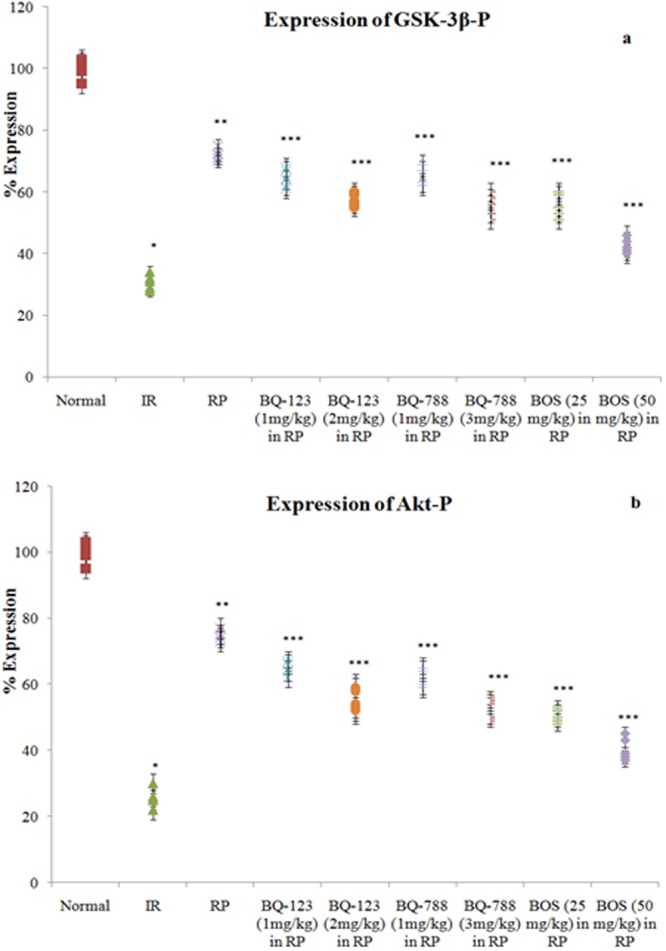
Consequences of I/R, RP and endothelin receptor antagonists on (**a**) levels of GSK-3β-P; (**b**) and Akt-P in the heart. *p < 0.05 *vs*. normal; **p < 0.05 *vs*. I/R; ***p < 0.05 *vs*. RP. One way ANOVA was followed by Tukey’s test and statistic parameters for GSK-3β-P expression were n = 6, F(8,45) = 980.1, p < 0.0001 and for Akt-P expression were n = 6, F(8,45) = 1001.5, p < 0.0001.

**Figure 6:**
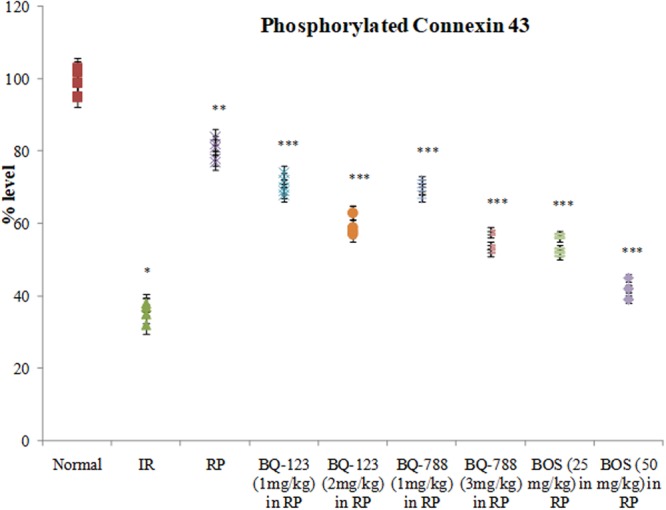
Consequences of I/R, RP and endothelin receptor antagonists on levels of phosphorylated connexin 43 in heart. *p < 0.05 *vs*. normal; **p < 0.05 *vs*. I/R; ***p < 0.05 *vs*. RP. One way ANOVA was followed by Tukey’s test and statistic parameters for P-connexin 43 were n = 6, F(8,45) = 787.8, p < 0.0001.

Along with myocardial injury, prolonged ischemia and reperfusion also led to significant alterations in the phosphorylated forms of Akt, GSK-3β and connexin 43 in heart. In comparison to normal hearts (not subjected to ischemia reperfusion episodes), there was a marked reduction in the levels of Akt-P, GSK-3β-P and P-connexin 43 in ischemia and reperfusion subjected isolated rat hearts **(**Figs 5a,b and 6**)**.

### Remote preconditioning decreased myocardial injury in ischemia reperfusion subjected hearts

Four short cycles of ischemia and reperfusion (5 minutes each) to the hind limb in the form of remote preconditioning protected the isolated rat hearts from prolonged ischemia-reperfusion injury. The release of specific biochemical markers of myocardial injury including LDH-1, CK-MB and cTnT was markedly reduced from remote preconditioning subjected hearts in comparison to ischemia reperfusion group **(**Figs 1a,b and 2a**)**. Moreover, there was an increase in the extent of TTC staining in remote preconditioning subjected hearts suggesting a decrease in myocardial infarction **(**Figs 2b and 3a**)**. There was significant decrease in capspase-3 activity and increase in Bcl-2 expression in hearts suggesting decrease in apoptosis **(**Fig. 4a,b**)**. Furthermore, remote prconditioning also led to improvement in heart contractility parameters including LVDP, dp/dt_max_, dp/dt_min_ and heart rate suggesting the functional improvement in heart **(**Tables 1, 2, 3 and 4**)**.

### Remote preconditioning modulated the levels of endothelin 1 in plasma and GSK-3β-P, Akt-P and P-connexin 43 in heart

Following four short episodes of hind limb ischemia and reperfusion (remote ischemic preconditioning stimulus), there was an increase in the levels of endothelin 1 in the plasma in comparison to ischemia-reperfusion group. However, there was no change in the levels of endothelin 2 and endothelin 3 **(**Fig. 3b**)**. Thirty minutes of global ischemia and 120 minutes of reperfusion decreased the levels of phosphorylated forms of Akt, GSK-3β and connexin 43 in the heart in comparison to normal hearts. However, remote preconditioning stimulus led to increase in the levels of Akt-P, GSK-3β-P and P-connexin 43 in the heart following prolonged ischemia and reperfusion **(**Figs 5a,b and 6**)**.

### Endothelin receptor antagonists modulated the cardioprotective effects of remote preconditioning

Pretreatment with BQ 123 (a selective inhibitor of ET_A_ receptors) and BQ 788 (a selective inhibitor of ET_B_ receptors) before remote preconditioning (thirty minutes prior to first episode of remote preconditioning stimulus) mitigated the cardioprotective effects of remote preconditioning. Administration of both BQ 123 (1 and 2 mg/kg) and BQ 788 (1 and 3 mg/kg) led to increase in the levels of LDH-1, CK-MB and cTnT in coronary perfusate in remote preconditioning groups **(**Figs 1a,b and 2a**)**. There was also an increase in myocardial infarct size **(**Fig. 3a**)**, increase in caspase-3 activity **(**Fig. 4a**)**, decrease in Bcl-2 expression **(**Fig. 4b**)** and decrease in contractility parameters **(**Tables 1, 2, 3 and 4**)**. Moreover, these pharmacological agents decreased the levels of Akt-P, GSK-3β-P and P-connexin 43 in the hearts of remote preconditioning group **(**Figs 5a,b and 6**)**. Pretreatment with bonsentan (a non-selective inhibitor of ET_A_ and ET_B_ receptors) mimicked the effects of BQ 123 and BQ 788 in remote preconditioning groups. However, the effects of bonsentan (25 mg/kg and 50 mg/kg) on remote preconditioning were much pronounced in comparison to BQ 123 and BQ 788. On other words, bonsentan abolished the effects of remote preconditioning to a greater extent as compared to BQ 123 and BQ 788. However, administration of vehicle (10% DMSO) did not influence the parameters of myocardial injury and kinases in heart in ischemia reperfusion subjected rats.

## Discussion

In the present study, we observed remote preconditioning reduced ischemia-reperfusion-induced cardiac injury, as indicated by reduction in the levels of LDH-1, CK-MB and cTnT in the coronary perfusate. These are specific biomarkers of cardiac injury and their release takes place during rupturing of cell membrane^34^. Remote preconditioning also attenuated ischemia-reperfusion induced increase in apoptosis as indicated by decrease in caspase-3 activity and increase in bcl-2 expression. It also reduced the extent of myocardial infarction (demonstrated by TTC staining) and improved myocardial contractility including LVDP, dp/dt_max_ dp/dt_min_ and heart rate. Remote preconditioning-induced myocardial protection is widely supported by preclinical as well as clinical studies^35,36^.

Moreover, remote preconditioning led to an increase in the levels of endothelin 1 in the blood, without altering the levels of endothelin 2 or endothelin 3. Endothelin-1 is the most important member of endothelin family and it is primarily released from the endothelium. It may be possible to suggest that during short cycles of ischemia and reperfusion to hind limb, the endothelial cells may respond to ischemia by releasing endothelin 1, which may travel to the heart as a humoral factor. There have been previous studies documenting that exogenous administration of endothelin 1 produces preconditioning like effects on heart and exerts cardioprotection^25,26^. Moreover, the protective effects of endothelin-1 pretreatment on neonatal rat cardiomyocytes against hypoxia-induced injury has also been reported^37^. Endothelin-1 has also been shown to promote the survival of mesenchymal stem cells^38^. The activation of endothelin 1 signaling pathway has been shown to exert cardioprotection against chronic intermittent hypoxia^24,39^. However, it is the first report documenting that endothelin 1 may be the possible humoral factor released during remote preconditioning to protect heart from sustained ischemia reperfusion injury in rats. The cardioprotective role of endothelin 1 in remote preconditioning was affirmed by pretreatment of rats with selective as well as non-selective ET_A_ and ET_B_ antagonists before remote preconditioning. Pretreatment with BQ-123 (a selective ET_A_ receptor antagonist); BQ-788 (a selective ET_B_ receptor antagonist) and bonsentan (nonselective ET_A_ and ET_B_ receptor antagonist) mitigated the protective effects of remote preconditioning on heart. It again emphasizes that remote preconditioning may increase the release of endothelin 1, which may travel to the heart through blood to activate myocardial ET_A_ and ET_B_ receptors to trigger cardioprotection. It is also worth mentioning that the attenuating effects of bonsentan on cardioprotection were significantly higher in comparison to BQ 123 and BQ 788. It suggests the crucial role of both ET_A_ and ET_B_ receptors in triggering cardioprotection during remote preconditioning.

To explore the molecular mechanisms involved in remote preconditioning, the expression levels of phosphorylated forms of pro-survival protein kinases Akt, GSK-3β along with gap junction protein, connexin 43 were measured in hearts. Remote preconditioning significantly restored the levels of Akt-P, GSK-3β-P and P-connexin 43 in ischemia-reperfusion subjected rats. Akt is a principal pro-survival protein kinase and Akt-P is the active form of this enzyme. Akt phosphorylates other downstream regulator enzymes including GSK-3β. In contrast to other enzymes, GSK-3β exists in the active form in the non-phosphorylated state and it is inactivated on phosphorylation^40^. Thus, an increase in Akt-P and GSK-3β-P levels during remote preconditioning suggests that there is an activation of Akt and inhibition of GSK-3β enzyme during remote preconditioning. Studies have documented that activation of pro-survival protein kinase Akt is important in remote preconditioning-induced cardioprotection^41^. There have been studies suggesting that inhibition of GSK-3β protects the heart from ischemia-reperfusion injury^40^. Moreover, an increase in the phosphorylation of GSK-3β and its inactivation has been documented in ischemic preconditioning as well as in remote preconditioning-induced cardioprotection^40,42,43^. Connexin 43 (also termed as Gap junction alpha-1 protein) is a critical component of gap junctions, which allow intercellular communication to take place and electrical impulses can pass between cells through these junctions^44^. There have been studies showing that an increase in gap junction coupling and preservation of phosphorylation of connexin 43 may participate in hypoxic preconditioning and remote preconditioning-induced cardioprotection^45,46^.

**Figure 7:**
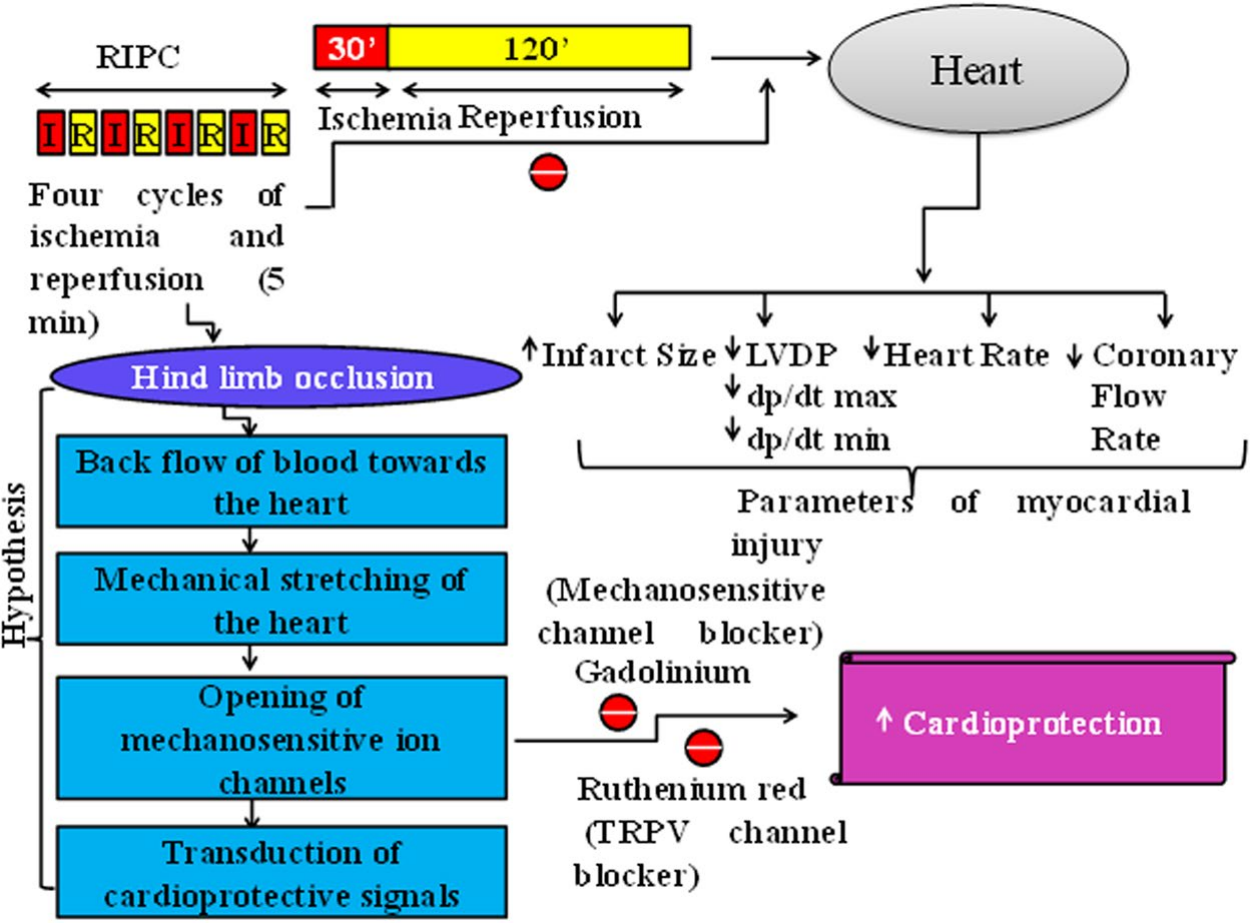
Proposed Hypothesis in remote preconditioning-induced cardioprotection. Four short episodes of ischemic episodes to hind limb may trigger the release of endothelin 1 from endothelium, which may act on ET_A_ and ET_B_ receptors. The activation of these receptors may increase phosphorylation of Akt, GSK-3β (inhibition of GSK-3β activity) and connexin 43 to induce cardioprotection. The selective (BQ 123 and BQ 788) as well as non-selective blockers (bonsentan) attenuated cardioprotective effects of remote preconditioning.

In the present study, pretreatment with BQ123, BQ788 and bonsentan attenuated the levels of pro-survival kinases Akt-P, GSK-3β-P and P-connexin 43. The effects of bonsentan were more significant as compared to BQ 123 and BQ 788, suggesting the key role of both ET_A_ and ET_B_ receptors in modulating the levels of downstream protein kinases and connexin 43. Therefore, it may be proposed that endothelin may activate both ET_A_ and ET_B_ receptors located on the heart to activate Akt, inhibit GSK-3β and increase P-connexin 43. There have been studies documenting that endothelin 1 may activate Akt phosphorylation and inhibit GSK-3β to produce its biological actions^47–49^. It is shown that the actions of endothelin-1 on astrocytes are dependent on connexin 43 levels^50^. Furthermore, there has been a study showing that endothelin 1 produces phosphorylation of Akt and GSK-3β through connexin 43 in cardiomyocytes^51^. Therefore, it may be proposed that remote preconditioning may increase the levels of endothelin 1 in the blood, which may subsequently activate myocardial ET_A_ and ET_B_ receptors to trigger cardioprotective signaling involving activation of Akt, inhibition of GSK-3β and preservation of connexin 43 phosphorylation **(**Fig. 7**)**.

### Strengths and Limitations of the Present Study

The major strength of this present work is that it is the first direct study showing an increase in the levels of endothelin 1 in the blood during remote preconditioning, which triggers Akt/GSK-3β and connexin 43 signaling cascade through ET_A_ and ET_B_ receptors to induce cardioprotection. However, the main limitation of the present study is that the interrelationship among Akt, GSK-3β and connexin 43 is not explored. Further studies are required to elucidate whether endothelin 1 directly increases the phosphorylation of connexin 43 or indirectly through Akt/GSK-3β.

## Conclusion

Remote preconditioning stimulus may activate the endothelial lining to release endothelin 1 in the blood, which may activate myocardial ET_A_ and ET_B_ receptors to trigger cardioprotection through connexin 43 and Akt/GSK-3β signaling pathway.

## References

[1] Przyklenk K, Sanderson TH, Huttemann M (2014). Clinical benefits of remote ischemic preconditioning: new insights… and new questions. Circ Res.

[2] Przyklenk K (2013). ‘Going out on a limb’: SDF-1alpha/CXCR4 signaling as a mechanism of remote ischemic preconditioning?. Basic Res Cardiol.

[3] Weselcouch EO (1995). Endogenous catecholamines are not necessary for ischaemic preconditioning in the isolated perfused rat heart. Cardiovasc Res.

[4] Xu MJ (2017). Immediate Early Response Gene X-1 (IEX-1) Mediates Ischemic Preconditioning-Induced Cardioprotection in Rats. Oxid Med Cell Longev.

[5] Dong W (2018). Adrenomedullin serves a role in the humoral pathway of delayed remote ischemic preconditioning via a hypoxia-inducible factor-1alpha-associated mechanism. Mol Med Rep.

[6] Yang, J. *et al*. Hypoxia Inducible Factor 1alpha Plays a Key Role in Remote Ischemic Preconditioning Against Stroke by Modulating Inflammatory Responses in Rats. *J Am Heart Assoc* **7** (2018). 10.1161/JAHA.117.007589 PMC5866324 29478025

[7] Gill R (2015). Remote ischemic preconditioning for myocardial protection: update on mechanisms and clinical relevance. Mol Cell Biochem.

[8] Lim SY, Yellon DM, Hausenloy DJ (2010). The neural and humoral pathways in remote limb ischemic preconditioning. Basic Res Cardiol.

[9] Diwan V, Kant R, Jaggi AS, Singh N, Singh D (2008). Signal mechanism activated by erythropoietin preconditioning and remote renal preconditioning-induced cardioprotection. Mol Cell Biochem.

[10] Liem DA, Verdouw PD, Duncker DJ (2003). Transient limb ischemia induces remote ischemic preconditioning *in vivo*. Circulation.

[11] Taliyan R, Singh M, Sharma PL, Yadav HN, Sidhu KS (2010). Possible involvement of alpha1-adrenergic receptor and K(ATP) channels in cardioprotective effect of remote aortic preconditioning in isolated rat heart. J Cardiovasc Dis Res.

[12] Wang Y, Xu H, Mizoguchi K, Oe M, Maeta H (2001). Intestinal ischemia induces late preconditioning against myocardial infarction: a role for inducible nitric oxide synthase. Cardiovasc Res.

[13] Miyauchi T (1989). Increased plasma concentrations of endothelin-1 and big endothelin-1 in acute myocardial infarction. Lancet.

[14] Pernow J, Wang QD (1997). Endothelin in myocardial ischaemia and reperfusion. Cardiovasc Res.

[15] Qiu S, Theroux P, Marcil M, Solymoss BC (1993). Plasma endothelin-1 levels in stable and unstable angina. Cardiology.

[16] Fox, B. M. *et al*. Acute Pressor Response to Psychosocial Stress Is Dependent on Endothelium-Derived Endothelin-1. *J Am Heart Assoc* **7** (2018). 10.1161/JAHA.117.007863 PMC5850198 29453306

[17] Zhai X, Leo MD, Jaggar JH (2017). Endothelin-1 Stimulates Vasoconstriction Through Rab11A Serine 177 Phosphorylation. Circ Res.

[18] Rubanyi GM, Polokoff MA (1994). Endothelins: molecular biology, biochemistry, pharmacology, physiology, and pathophysiology. Pharmacol Rev.

[19] Kobayashi T (1999). Expression of endothelin-1, ETA and ETB receptors, and ECE and distribution of endothelin-1 in failing rat heart. Am J Physiol.

[20] Maguire JJ, Davenport AP (2015). Endothelin receptors and their antagonists. Semin Nephrol.

[21] Cadenas S (2018). ROS and redox signaling in myocardial ischemia-reperfusion injury and cardioprotection. Free Radic Biol Med.

[22] Meldrum DR (1996). Cardiac preconditioning with calcium: clinically accessible myocardial protection. J Thorac Cardiovasc Surg.

[23] Richard V (1994). Role of endogenous endothelin in myocardial and coronary endothelial injury after ischaemia and reperfusion in rats: studies with bosentan, a mixed ETA-ETB antagonist. Br J Pharmacol.

[24] Wang, J. W. *et al*. Endothelin-1 and ET receptors impair left ventricular function by mediated coronary arteries dysfunction in chronic intermittent hypoxia rats. *Physiol Rep* **5** (2017). 10.14814/phy2.13050 PMC5256153 28057852

[25] Bugge E, Ytrehus K (1996). Endothelin-1 can reduce infarct size through protein kinase C and KATP channels in the isolated rat heart. Cardiovasc Res.

[26] Gourine AV (2005). Endothelin-1 exerts a preconditioning-like cardioprotective effect against ischaemia/reperfusion injury via the ET(A) receptor and the mitochondrial K(ATP) channel in the rat *in vivo*. Br J Pharmacol.

[27] Helmy MM, Helmy MW, Abd Allah DM, Abo Zaid AM, Mohy El-Din MM (2014). Selective ET(A) receptor blockade protects against cisplatin-induced acute renal failure in male rats. Eur J Pharmacol.

[28] Moldes O (2012). Neuroprotection afforded by antagonists of endothelin-1 receptors in experimental stroke. Neuropharmacology.

[29] Afyouni NE (2015). Preventive Role of Endothelin Antagonist on Kidney Ischemia: Reperfusion Injury in Male and Female Rats. Int J Prev Med.

[30] Kharbanda RK (2002). Transient limb ischemia induces remote ischemic preconditioning *in vivo*. Circulation.

[31] Langendorff, O. Untersuchungen amuber lebenderer saugethierherzen. *Pfluger*. *Arch*. *Gesmate*. *Physio*. **61** (1885).

[32] Guo J (2013). Coptisine protects rat heart against myocardial ischemia/reperfusion injury by suppressing myocardial apoptosis and inflammation. Atherosclerosis.

[33] Grunenfelder J (2001). Upregulation of Bcl-2 through caspase-3 inhibition ameliorates ischemia/reperfusion injury in rat cardiac allografts. Circulation.

[34] Kemp M, Donovan J, Higham H, Hooper J (2004). Biochemical markers of myocardial injury. Br J Anaesth.

[35] Sharma R, Randhawa PK, Singh N, Jaggi AS (2016). Possible role of thromboxane A2 in remote hind limb preconditioning-induced cardioprotection. Naunyn Schmiedebergs Arch Pharmacol.

[36] Thomas KN, Cotter JD, Williams MJ, van Rij AM (2016). Repeated Episodes of Remote Ischemic Preconditioning for the Prevention of Myocardial Injury in Vascular Surgery. Vasc Endovascular Surg.

[37] Pan YX, Lin L, Yuan WJ, Tang CS (2003). [Preventive effect of endothelin-1 pretreatment on hypoxia-induced injury in cultured neonatal rat cardiomyocytes]. Sheng Li Xue Bao.

[38] Pourjafar M (2016). Cytoprotective effects of endothelin-1 on mesenchymal stem cells: an *in vitro* study. Clin Exp Pharmacol Physiol.

[39] Wang N (2017). Tanshinone IIA protects against chronic intermittent hypoxia-induced myocardial injury via activating the endothelin 1 pathway. Biomed Pharmacother.

[40] Rahman S (2011). Phosphorylation of GSK-3beta mediates intralipid-induced cardioprotection against ischemia/reperfusion injury. Anesthesiology.

[41] Slagsvold KH (2014). Remote ischemic preconditioning preserves mitochondrial function and activates pro-survival protein kinase Akt in the left ventricle during cardiac surgery: a randomized trial. Int J Cardiol.

[42] Randhawa PK, Jaggi AS (2017). Investigating the involvement of glycogen synthase kinase-3beta and gap junction signaling in TRPV1 and remote hind preconditioning-induced cardioprotection. Eur J Pharmacol.

[43] Tong H, Imahashi K, Steenbergen C, Murphy E (2002). Phosphorylation of glycogen synthase kinase-3beta during preconditioning through a phosphatidylinositol-3-kinase–dependent pathway is cardioprotective. Circ Res.

[44] Severs NJ, Bruce AF, Dupont E, Rothery S (2008). Remodelling of gap junctions and connexin expression in diseased myocardium. Cardiovasc Res.

[45] Brandenburger T (2014). Remote ischemic preconditioning preserves Connexin 43 phosphorylation in the rat heart *in vivo*. J Transl Med.

[46] Rath G (2012). Vascular hypoxic preconditioning relies on TRPV4-dependent calcium influx and proper intercellular gap junctions communication. Arterioscler Thromb Vasc Biol.

[47] Cianfrocca R (2010). Beta-arrestin-1 mediates the endothelin-1-induced activation of Akt and integrin-linked kinase. Can J Physiol Pharmacol.

[48] D’Antoni S, Ranno E, Spatuzza M, Cavallaro S, Catania MV (2017). Endothelin-1 Induces Degeneration of Cultured Motor Neurons Through a Mechanism Mediated by Nitric Oxide and PI3K/Akt Pathway. Neurotox Res.

[49] Han MM, Yang CW, Cheung CW, Li J (2018). Blockage of spinal endothelin A receptors attenuates bone cancer pain via regulation of the Akt/ERK signaling pathway in mice. Neuropeptides.

[50] Tabernero A, Jimenez C, Velasco A, Giaume C, Medina JM (2001). The enhancement of glucose uptake caused by the collapse of gap junction communication is due to an increase in astrocyte proliferation. J Neurochem.

[51] Ishikawa S (2012). Role of connexin-43 in protective PI3K-Akt-GSK-3beta signaling in cardiomyocytes. Am J Physiol Heart Circ Physiol.

